# Comparative On-Farm Welfare Assessment of Sheep in Extensive, Semi-Extensive, and Semi-Intensive Systems

**DOI:** 10.3390/vetsci13040329

**Published:** 2026-03-28

**Authors:** Snežana Paskaš, Ivan Pihler, Marija Pajić, Elmin Tarić, Miloš Dimitrijević, Katarina Pajić, Zsolt Becskei

**Affiliations:** 1 Department of Animal Science, Faculty of Agriculture, University of Novi Sad, 21000 Novi Sad, Serbia; snezana.paskas@proton.me (S.P.); ivan.pihler@stocarstvo.edu.rs (I.P.); 2 Department of Veterinary Medicine, Faculty of Agriculture, University of Novi Sad, 21000 Novi Sad, Serbia; marija.pajic@polj.edu.rs (M.P.); dimitrijevic.dvm96@gmail.com (M.D.); katarina.cobanov20@gmail.com (K.P.); 3 Department of Animal Breeding and Genetics, Faculty of Veterinary Medicine, University of Belgrade, 11000 Belgrade, Serbia; etar1989@yahoo.com

**Keywords:** sheep welfare, housing conditions, management practices, health indicators

## Abstract

Sheep welfare outcomes vary depending on production systems, breeds, and environmental conditions. This study examined the effects of extensive, semi-extensive, and semi-intensive sheep production systems on animal welfare in Serbia, using the AWIN Welfare Protocol to evaluate 30 farms. Welfare indicators were categorised into resource-based, management-based, and animal-based metrics. The results indicated that there was no significant difference in space allowance among the production systems (*p* > 0.05). This suggests that the space provided was adequate for semi-intensive farms and suitable for both semi-extensive and extensive farms. However, management practices showed significant variations (*p* < 0.05), indicating diverse impacts on sheep welfare. No ocular discharge or stereotypic behaviours were observed, while respiratory issues, social withdrawal, and excessive itching were found to have a very low prevalence across all farms. The primary welfare concern identified in the extensive farms was the use of painful mutilations. Semi-extensive and semi-intensive farms had significantly higher rates of tail docking (*p* < 0.05) and poorer fleece cleanliness. These findings highlight the necessity of addressing the root causes of poor welfare to improve sheep welfare standards. Therefore, achieving sustainable welfare outcomes requires an integrated approach that combines genetic suitability, adequate housing, and effective management practices.

## Simple Summary

Comparing sheep production systems is crucial for identifying their strengths and weaknesses. Research on sheep welfare across different production systems aims to understand how management practices, housing, and feeding conditions influence animal health and well-being. The findings indicate that welfare outcomes vary among systems; however, these differences are primarily driven by management practices, environmental exposure, and breed adaptation. Semi-intensive farms showed advantages in structured health control and nutrition management, whereas extensive systems performed better in terms of behavioural expression and the resilience of local breeds. Welfare risks were system-specific, particularly regarding lameness management and nutritional variability. This research provides evidence on system-specific welfare strengths and risks, supporting the development of more targeted and sustainable sheep farming practices.

## 1. Introduction

The welfare of sheep is vital for sustainable, efficient, and productive farming systems, particularly in regions where grazing contributes to the preservation of biodiversity and traditional landscapes [1,2]. Maintaining high welfare standards is essential given their legal and ethical importance and their direct economic implications [3]. Growing societal concern for animal welfare has driven improvements in methods for assessing the well-being of small ruminants [4]. However, research on sheep welfare continues to progress more slowly than in other ruminants, such as cattle, due to the species’ specific physiological characteristics and the predominance of extensive production systems [1]. This gap limits the adoption of welfare-enhancing practices as continuous monitoring remains challenging. A key issue is the lack of validated indicators that accurately capture positive welfare experiences in farm animals, particularly in extensively reared species such as sheep [5]. Rivero and Lee [2] highlighted that welfare indicators often focus on negative aspects of nutrition, the environment, and health, such as poor feed quality, injuries, and disease, because of the difficulty of measuring positive states. However, these indicators do not adequately address broader behavioural and affective components, including the expression of rewarding behaviours. Furthermore, assessing positive welfare in extensively reared animals presents practical challenges. Currently, only a limited number of assessment protocols are available, most of which are designed primarily for meat-producing sheep. In addition, many of these protocols are adapted to extensive systems and may not fully address all five freedoms of animal welfare [6]. Nevertheless, the Animal Welfare Indicators (AWIN) protocol for sheep represents a valuable tool, as it incorporates both flock-level and individual-based indicators, enabling the identification of management deficiencies and the development of targeted interventions [7].

Parés et al. [8] identified the production system as a key factor influencing welfare outcomes in sheep, although seasonality, breed, and flock size also affect specific indicators. Extensive systems for small ruminants are highly heterogeneous and are typically located on marginal grazing lands. These systems are often characterised by harsh climatic conditions, limited infrastructure, and restricted management capacity. Consequently, they face multiple challenges, including nutritional deficiencies, heat stress, neonatal losses, metabolic disorders, parasitism, and associated welfare concerns [9,10]. In contrast, intensive systems focus on microclimate control and optimised housing design, which can help prevent overcrowding, reduce aggressive behaviour, and limit environmental and health-related risks, such as udder disorders [1].

The welfare of sheep is also strongly influenced by the stockperson’s competence. In extensive systems, human–animal interactions are less frequent and may be associated with increased reactivity during handling. Despite these challenges, extensive dairy sheep systems play an important role in rural development by providing income and employment in marginal areas with limited economic alternatives [11,12]. Moreover, mixed grazing systems involving sheep, goats, and cattle can improve the efficiency of pasture utilisation [12,13]. Wróbel et al. [14] demonstrated that pasture-based feeding enhances behavioural expression and supports positive mental states by allowing animals to interact with a more natural environment. However, welfare outcomes in grazing systems remain highly dependent on management quality as environmental factors strongly influence nutrition, health, and overall well-being. Under certain conditions, more controlled production systems may offer advantages [2,4].

Despite increasing global attention to animal welfare, there is still limited information on the welfare of sheep raised under different production systems in many countries, including Serbia. Understanding how these systems influence welfare outcomes is essential for improving management practices, promoting sustainability, and meeting societal expectations. In Serbia, consumer awareness of animal welfare has increased, with pasture-based dairy systems often perceived as more favourable [15]. Although a legal framework has been in place for over a decade, farmers continue to face challenges in achieving high welfare standards in small ruminant production [3]. Furthermore, Phythian et al. [16] reported that many Serbian farmers intend to expand their production, which may create opportunities for welfare improvement, provided that adequate financial and technical support is available.

The objective of this study was to evaluate and compare sheep welfare across the three most common sheep farming systems in Serbia: extensive, semi-extensive, and semi-intensive. This evaluation utilised indicators based on resources, management practices, and animal welfare, including behavioural measures related to discomfort, pain, disease, and fear. It was hypothesised that welfare outcomes would differ among production systems, primarily reflecting differences in management practices and system-specific characteristics.

## 2. Materials and Methods

### 2.1. Survey and Characteristics of Farms

Farming conditions, management systems, and sheep breeds vary significantly across Serbia. From January to October 2025, data were collected from thirty sheep farms representing the three most common production systems. These farms were categorised as extensive, semi-extensive, or semi-intensive based on resource utilisation and management practices, reflecting differences in feeding, housing, and farm inputs. A total of ten farms were analysed within each production system. The majority of sheep were raised for meat production, while a smaller proportion were managed under dual-purpose systems (Table 1). The farms were located in three regions: the Pešter Plateau in southern Serbia, Vojvodina Province, and the Mačva District in central Serbia. In terms of altitude, ten farms were situated in mountainous areas, four in hilly regions of central Serbia, and sixteen in lowland areas. Farms were selected based on production system, farm size, and availability. Welfare assessment methods were applied to adult female sheep and did not require specialised equipment.

### 2.2. The Design of the Questionnaire

The methodology and questionnaire were developed based on the AWIN welfare protocol for sheep [17] and the assessment protocol for cattle [18]. The AWIN protocols (Animal Welfare Indicators Project, EU FP7) are standardised, animal-based tools for welfare assessment, developed for species such as horses, donkeys, goats, sheep, and turkeys. These protocols provide practical, science-based measures of physical and behavioural welfare and are widely recognised as reliable frameworks in animal welfare science [19]. The animal welfare protocol for dairy cattle, based on the Five Freedoms, was adapted for sheep. The questionnaire consisted of 75 questions, including 15 specifically related to milk-producing sheep. Farms were evaluated according to four key welfare principles: adequate housing, proper management, good health (animal-based indicators), and appropriate behaviour, defined as the expression of natural, species-specific behaviours essential for maintaining good welfare [20]. This also included ensuring the absence of tethered or isolated animals and enabling access to grazing.

The questionnaire was divided into three sections:

• Identification data;
• Management- and resource-based indicators;
• Animal-based indicators (group- and individual-level observations).

The identification section included general farm information, such as sheep breeds, farm size, production system, and selected farmer-related management practices (Table 1). The second section focused on management practices and resource-based indicators, particularly housing conditions. This included housing type, bedding quality and quantity, dryness, space allowance, water supply characteristics, and availability of handling facilities. In addition, farmers were surveyed regarding feeding management practices. Specifically, the section also included questions on lamb mortality (at birth and before weaning), castration and tail docking practices, flock health planning, and the mixing of animals of different ages and sizes.

### 2.3. Assessment of the Sheep Welfare

Indicators were analysed at both the individual and group levels, including environmental aspects, to provide a comprehensive welfare evaluation. A total of 19 welfare indicators were assessed, including nine group-level and ten individual-level indicators. The AWIN protocol follows a two-stage approach. The first stage consists of a preliminary flock-level assessment, followed by a more detailed individual-level evaluation. Initially, a rapid group-level screening is performed for each pen, followed by in-depth observation of individual animals. On farms with multiple pens, additional pens were included in the assessment. The first stage focuses on rapid screening using reliable animal-based indicators that require minimal or no handling. The second stage is applied if the farm is identified as non-compliant with animal welfare standards or if specific indicators fall within the lowest 5% of the reference population. This approach minimises animal stress while reducing the time required for assessment [17]. The number of individually assessed animals depended on the number of sheep present in each pen. Farm-level prevalence was calculated as the proportion of affected animals relative to the total number assessed. Group-level prevalence was calculated as the mean prevalence across farms within the same production system, following standard epidemiological principles. Welfare assessments were conducted by two assessors: one experienced in farm animal welfare assessment and a second assessor trained before the study. In total, 1378 animals were evaluated across all farms. The assessment began with flock-level observation for clinical signs of disease, lameness, and respiratory symptoms, followed by clinical examination of randomly selected animals.

### 2.4. Assessment of Sheep Behaviour

Behavioural assessment focused on indicators of fear and the human–animal relationship, using animal-based measures. Social withdrawal, stereotypic behaviour, qualitative behaviour assessment, and the human approach test were conducted according to the AWIN protocol [17]. Social withdrawal was assessed through 15-min observations, recording behaviours such as isolation from the group or reduced responsiveness. Stereotypic behaviours were also recorded, including repetitive and non-functional actions such as head turning, upward gazing, or wool pulling. The human–animal relationship was evaluated by observing sheep responses when the primary stockperson entered the pen and handled animals. Responses were categorised into three levels: “pass freely”, “pass timidly”, and “show fear in the presence of humans” [17]. Sheep that approached within 2 m were considered sociable and restless; those between 2 and 5 m were categorised as neutral and cautious, while those over 5 m were viewed as fearful and aversive. An average score was calculated for each farm. Total observation time per farm ranged from 40 to 70 min.

### 2.5. Statistical Analysis

Descriptive statistics were used to summarise the data and evaluate differences among production systems. Measures of central tendency (mean) and dispersion (minimum, maximum, percentage, and standard deviation) were calculated. Categorical data were analysed using the Pearson chi-squared test. The normality of animal-based welfare indicators was assessed using the Shapiro–Wilk test, and homogeneity of variances was tested using Levene’s test. When parametric assumptions were met, differences among production systems were analysed using one-way ANOVA followed by Tukey’s post hoc test. For non-normally distributed data, the Kruskal–Wallis test was applied. Statistical significance was set at *p* < 0.05. Principal component analysis (PCA) was performed separately for group-level and behavioural indicators to identify structural patterns and relationships among variables. Statistical analyses were conducted using R software (version 4.4.2; R Core Team, Vienna, Austria) and Microsoft Excel.

## 3. Results

### 3.1. Resource-Based Indicators

The results of the resource-based indicators are presented in Table 2. Most farms across all three production systems provided outdoor access through exterior pens. Semi-intensive farms showed significantly higher use of automatic drinkers (*p* < 0.05). Significant differences were also observed in drinker cleanliness. Semi-intensive and semi-extensive farms had the highest proportion of dirty drinkers (30%). However, the cleanliness of automatic drinkers was generally satisfactory. Straw bedding was used on all farms, with significantly better cleanliness observed in semi-intensive systems (*p* < 0.05). Space allowance was considered satisfactory in extensive and semi-extensive systems and adequate in semi-intensive systems. No tethered animals were observed on any of the farms.

Significant differences were also observed in pasture type (*p* < 0.05). Extensive farms primarily used unfenced hill pastures (70%), followed by fenced unimproved pastures (20%) and unfenced lowland pastures (10%). Semi-extensive farms utilised unfenced hill pastures (20%), fenced unimproved pastures (10%), and fenced lowland pastures (10%). Pasture systems are rarely utilised on these farms because they prioritise meat production using imported breeds. In contrast, semi-intensive farms relied on fenced improved pastures (30%), fenced unimproved pastures (40%), and, to a lesser extent, unfenced lowland pastures (10%).

### 3.2. Management-Based Indicators

The results of the management-based indicators are presented in Table 3. Significant differences in management practices were observed on farms across production systems (*p* < 0.05). Extensive farms primarily relied on pasture-based systems, with sheep grazing for an average of 252 days per year. Mixed grazing with cattle or other livestock was common in these systems, reported on 80% of extensive farms. All farms sheared sheep once annually, typically between April and June, although some farms, particularly those located in lowland areas of Vojvodina and raising imported breeds, began shearing as early as March. Indoor-based systems (semi-extensive and semi-intensive) more frequently applied earlier shearing practices, whereas autochthonous breeds were generally shorn later, in May and June.

As shown in Table 3, disease occurrence differed significantly among production systems (*p* < 0.05). Lameness and fasciolosis were more prevalent on semi-extensive farms, whereas diseases were more evenly distributed across extensive and semi-intensive systems. On extensive farms, the most frequently reported diseases in rams included lameness, pneumonia, sarcoptic mange, fasciolosis, and oestrosis. Semi-extensive farms most commonly reported pneumonia, lameness, urolithiasis, and fasciolosis, while semi-intensive farms reported pneumonia, urolithiasis, and parasitic infections. Diarrhoea was the most common condition observed in lambs across all production systems. Contagious ecthyma was reported on four farms, while pneumonia in lambs was recorded on one farm. Coccidiosis was observed on three semi-intensive farms, pneumonia on two, and contagious ecthyma on one. Lamb castration without the use of pain relief was reported on eight extensive and two semi-extensive farms. Bedding management differed significantly among systems (*p* < 0.05), with semi-intensive farms replacing bedding more frequently and performing more regular hoof inspection and trimming.

Feeding practices also varied significantly. Semi-extensive and semi-intensive farms predominantly used alfalfa hay, whereas extensive farms relied mainly on meadow hay. Semi-intensive systems incorporated higher proportions of corn silage and used significantly greater amounts of concentrate mixtures (*p* < 0.05). In contrast, extensive farms more often produced their own on-farm concentrate mixtures. Additional feed resources, used in smaller quantities, included aftermath pasture, soybean straw, clover, rye straw, sugar beet pulp, sudangrass, and fruit pomace.

#### Management System and Production Data for Dairy Ewes

**Figure 1:**
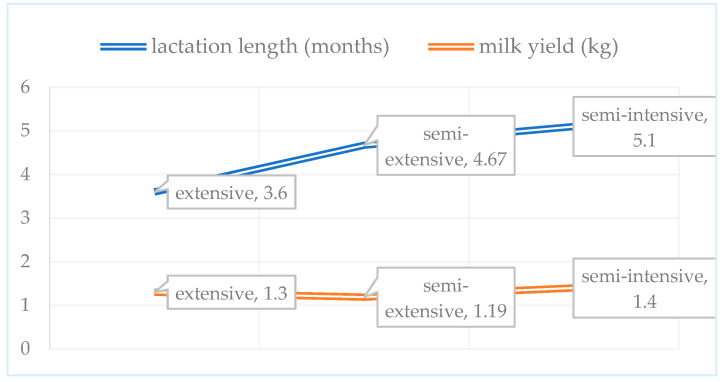
Lactation length and milk yield of dairy ewes.

Figure 1 presents the average lactation duration and milk yield per ewe across different production systems. The relatively low milk yield is primarily attributed to the predominance of local breeds with lower production potential. On all farms, lambs remained with their dams after birth, and milking was performed manually. The average milking period ranged from 3.6 months in extensive systems to five months in semi-intensive systems. The survey results indicated no substantial differences among production systems in general hygiene and sanitary practices. The use of gloves (nitrile or latex) and post-milking teat dipping was not reported on any farm. However, semi-intensive farms were distinguished by the use of udder washing procedures and single-use towels.

### 3.3. Animal-Based Indicators (Group-Level)

**Figure 2:**
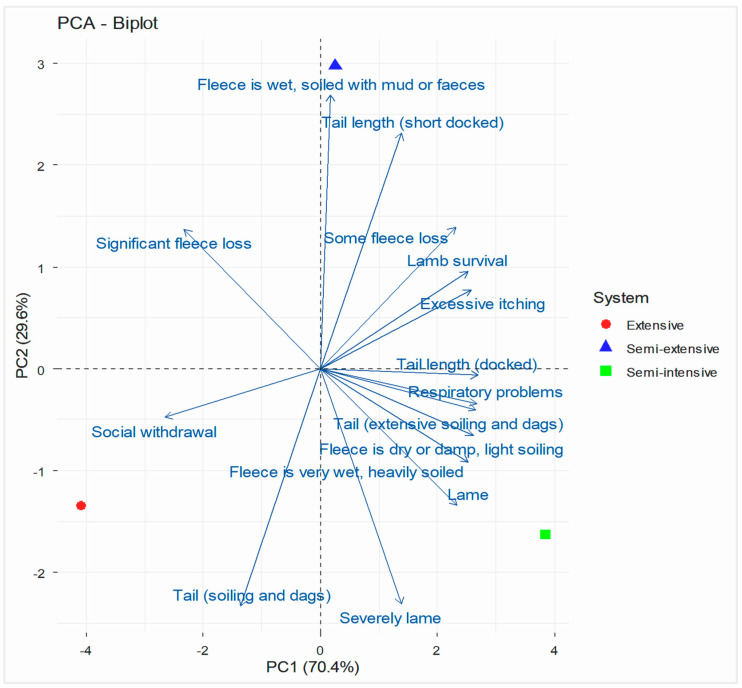
PCA biplot of sheep farms based on individual-based welfare indicators (group level).

Animal-based indicators (group-level) are shown in Figure 2. Principal component analysis (PCA) revealed distinct structural differences among farms. The first principal component (PC1) explained 24.5% of the total variance, while the second principal component (PC2) accounted for 22.5%, resulting in a total explained variance of 47.0%. PC1 represented the main axis of variation, separating farms based on health and management. For PC1, higher loadings were associated with indicators such as respiratory issues, excessive itching, social withdrawal, and poor fleece condition (wet or heavily soiled), indicating that farms with higher PC1 scores were considered to be in poorer health and welfare. Conversely, lower PC1 scores indicated fewer clinical signs and better fleece condition. The second principal component (PC2) further differentiated farms according to fleece condition and tail characteristics. For PC2, negative loadings were observed for fleece loss and short-docked tails, suggesting that animals with lower PC2 scores were more affected by fleece damage and docking practices. For PC3 (20.77%), higher scores were linked to tail soiling and lameness, implying animals with elevated PC3 values were more prone to hygiene issues and mobility problems.

Consequently, Cluster 1 consists of farms that experience a higher prevalence of health and hygiene-related issues. In contrast, Cluster 2 includes farms with specific management-related issues, such as tail docking, fleece loss, and severe lameness. These farms display a distinct welfare risk profile. Overall, the clusters highlight different welfare challenges and propose targeted management interventions tailored to each production system.

Additionally, no stereotypic behaviours or excessively wet fleece were observed across production systems. The prevalence of social withdrawal, excessive itching, respiratory problems, and very dirty or wet fleece was consistently low, indicating generally satisfactory behavioural and health conditions. Short-docked tails were not recorded on extensive farms, suggesting that tail docking is uncommon in these systems, whereas this practice was more frequent in semi-extensive farms. In contrast, semi-intensive farms showed a less favourable trend in lamb survival (2.99%, 5.27%, and 5.57%, for extensive, semi-extensive, and semi-intensive farms, respectively).

### 3.4. Animal-Based Indicators (Individual-Level)

The results of the individual-level animal-based indicators are presented in Table 4. Extensive farms showed a significantly lower prevalence of mild lameness (*p* < 0.05) compared to the other production systems. They also had fewer cases of very thin and very fat ewes, as well as a lower prevalence of hoof overgrowth. In contrast, semi-extensive farms exhibited the highest prevalence of injuries (*p* < 0.05), particularly affecting the head, body, and legs. Although no cases of ocular discharge were recorded, semi-intensive farms showed a significantly lower prevalence of nasal discharge, but a higher occurrence of hoof overgrowth and slightly poorer fleece cleanliness. Semi-extensive farms also showed a slightly higher prevalence of mastitis (both mild and severe); however, these differences were not statistically significant (*p* > 0.05).

### 3.5. Sheep Behaviour Assessment

**Figure 3:**
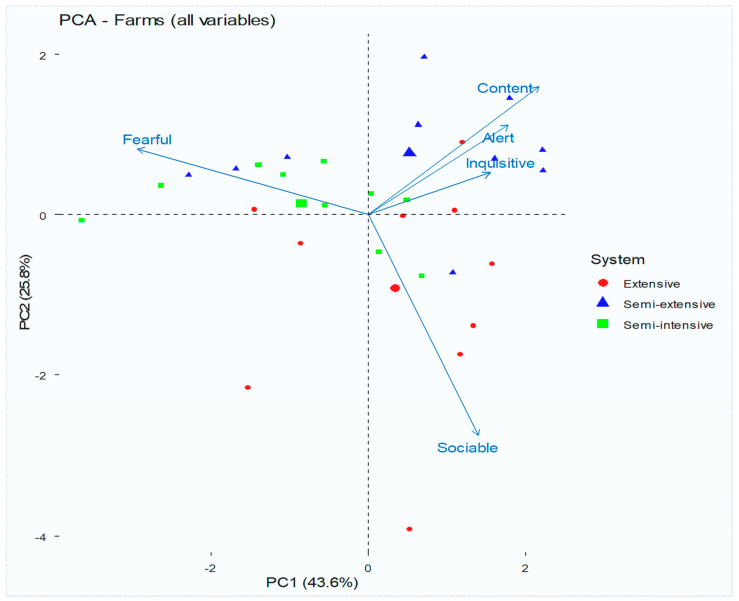
PCA biplot of sheep farms based on behaviour welfare indicators.

Sheep Behaviour Assessment are shown in Figure 3. Principal component analysis of individual-level behavioural indicators revealed a strong data structure, with the first principal component (PC1) explaining 73.0% of the total variance and the second component (PC2) accounting for 22.9%. Together, these two components explained 95.8% of the total variance. PC1 primarily separated animals exhibiting positive behavioural responses (content, alert, inquisitive) from those characterised by fearfulness, reflected sociable and content animals, indicating that PC1 represents a calm–fearful axis. Behaviour is strongly structured (PC1) but not exclusively determined by the production system. For PC2, positive loadings were linked to sociability, whereas negative loadings were associated with content and inquisitive behaviours, suggesting variation in social engagement. For PC3 (3.67%), inquisitiveness showed strong positive loadings, whereas content showed negative loadings, indicating that PC3 differentiates between exploratory and passive states. Finally, for PC4 (0.52%), fearful, sociable, and content also loaded positively, suggesting that this component reflects mixed dimensions of arousal and social interaction.

The clustering patterns reflect distinct behavioural profiles. Positive behavioural expressions characterise Cluster 1, while Cluster 2 is associated with fear-related responses. While some grouping by production system was noticed, the overlap among these systems suggests that behavioural outcomes are affected not only by the type of production but also by specific factors on the farm, including management practices, human–animal interactions, and breed characteristics. Additionally, the prevalence of fearfulness was significantly lower (*p* < 0.05) on extensive farms, which may be partly attributed to breed differences. These farms predominantly raised the native Sjenička breed, whereas semi-intensive and semi-extensive farms more frequently used imported breeds such as Île de France and Württemberg, which are generally more reactive and less sociable.

## 4. Discussion

According to Dwyer [21], animal welfare can be conceptualised through three interrelated dimensions: biological functioning, natural living conditions, and affective state. In this context, welfare reflects not only measurable health and productivity parameters but also the animal’s subjective experience while coping with environmental challenges [3]. Koçak et al. [22] emphasised that ensuring high-quality nutrition, adequate housing, and appropriate handling practices is fundamental to maintaining sheep welfare. Similarly, Sablik et al. [23] highlighted the importance of well-designed housing facilities that provide a suitable microclimate and allow unrestricted access to pasture. The present findings indicate that Serbian small-scale ruminant producers invest limited resources in housing infrastructure, which is consistent with previous observations on goat farms [3]. In extensive systems, modest housing conditions may compromise welfare during winter, particularly in mid-winter when animals are often in poorer body condition [24]. This constraint is partly mitigated in spring, when sheep spend most of their time grazing. Consistent with Garmendia et al. [12], grazing-based dairy sheep systems offer ecological and socioeconomic advantages while supporting behavioural welfare through the expression of natural behaviours. However, the present results demonstrate that grazing alone does not ensure optimal welfare outcomes. Management quality and environmental variability remain key determinants of nutritional balance, health status, and overall resilience, as also reported by Rivero and Lee [2] and Keyon and Cranston [25]. Therefore, the welfare performance of extensive systems should not be idealised but rather evaluated within the specific management context. Goddard [26] noted that the success of extensive or ranch-based systems depends on careful breed selection that ensures compatibility between genotype and environmental and management conditions. The findings also demonstrated that semi-intensive farms provided more adequate space allowances, averaging 1.78 m^2^ per animal. This stocking density appears sufficient to ensure comfort during resting, allowing animals to lie down simultaneously in clean and dry areas. These results are consistent with those reported by Koçak et al. [22], who documented similar space allowances (1.7 m^2^ per animal) on semi-extensive sheep farms in Turkey. Greater space availability, particularly when combined with access to outdoor areas, may enhance social interactions and feeding behaviour, potentially contributing to improved production performance. Ewes are particularly sensitive to stocking density, highlighting the importance of maintaining adequate space throughout different production stages [27]. Parés et al. [8] reported that bedding conditions significantly influence sternum cleanliness, proposing this parameter as a reliable indicator of resting comfort. In agreement with these findings, the present results identified bedding quantity and cleanliness as key determinants of welfare on semi-intensive farms, further emphasising the importance of resource-based indicators in welfare assessment.

Extensive evidence indicates that effective flock management is essential for maintaining sheep welfare while ensuring biological efficiency [1,28]. Management practices should prioritise the ethological and physiological needs of animals [29]. While some husbandry procedures, such as ectoparasite control, are minimally invasive, others, particularly castration and related mutilations, pose significant welfare concerns [24]. In the present study, painful mutilation practices were reported on 80% of extensive farms and 20% of semi-extensive farms, indicating a substantial welfare risk for lambs and supporting concerns raised by Koçak et al. [22]. Notably, management structure differed markedly across systems. All semi-intensive farms implemented herd health and welfare plans and conducted regular veterinary inspections, whereas only 60% of semi-extensive and 20% of extensive farms maintained written health plans. This gradient suggests that formalised management practices are associated with system intensification and may represent a key factor in preventive welfare management.

Sheep are ruminants frequently raised in marginal environments characterised by seasonal variability and limited supplementary feeding. Under such conditions, animals may experience both short- and long-term undernutrition, which can adversely affect productivity, reproductive performance, and overall welfare [25]. In the present study, semi-intensive farms relied on forage-based diets supplemented with concentrates and implemented management practices aimed at balancing productivity with welfare considerations. This hybrid approach integrates elements of traditional grazing and controlled feeding systems, potentially reducing nutritional fluctuations. In contrast, extensive farms primarily depended on grazing, with minimal supplementation and occasional use of lower-quality concentrates. Semi-extensive farms, however, neither fully utilise grazing potential nor consistently provide higher-quality concentrate supplementation. This intermediate system may therefore lack the structural advantages of both extensive and semi-intensive systems, potentially leading to less stable nutritional management. Regarding nutritional welfare indicators, variability in body condition score (BCS) was most pronounced in semi-intensive and semi-extensive systems, where both over-conditioned and under-conditioned ewes were observed. This pattern suggests inconsistencies in ration formulation or feeding management. In comparison, Parés et al. [8] reported higher average BCS values in intensive systems, reflecting more controlled nutritional management. These findings indicate that nutritional control, rather than system classification alone, is a key determinant of welfare outcomes. Furthermore, high neonatal lamb losses can be detrimental, as they reduce the availability of replacement females and compromise long-term flock sustainability. Early weaning represents an additional welfare concern in dairy systems; separation of lambs from their dams and artificial rearing may induce stress responses in both ewes and offspring, potentially impairing lamb growth, health, and survival [22]. However, the present findings challenge some of these assumptions. Early weaning was generally not practised, even on farms combining milk and meat production. Moreover, extensive systems exhibited the lowest lamb mortality rates. This outcome may reflect the use of locally adapted breeds with greater resilience to environmental constraints and lower nutritional requirements.

Hoof condition is widely recognised as an important welfare indicator, particularly in relation to locomotion and pain associated with lameness [4]. Regular foot inspection enables early detection and treatment of lesions that may predispose animals to lameness [30]. Damaged, misshapen, or overgrown claws can directly impair gait and mobility [31]. In the present study, semi-intensive farms performed routine foot checks more frequently (80%) than semi-extensive (70%) and extensive systems (20%), indicating a clear gradient in preventive management intensity. While claw trimming supports optimal hoof conformation and locomotor function, its necessity varies across production systems. In pastoral systems, natural wear on hard or stony terrain may reduce the need for trimming [30]. Conversely, wet and soft ground conditions limit natural abrasion, predisposing hooves to overgrowth and subsequent lameness [31,32]. Nutritional deficiencies in low-input systems may further compromise hoof horn quality, increasing susceptibility to injury [30,32]. Importantly, Smith et al. [32] demonstrated that hoof overgrowth is not necessarily a predisposing factor for lameness but may develop as a consequence of altered weight-bearing once animals become lame. This distinction is critical when interpreting hoof overgrowth as a welfare indicator. Koçak et al. [22] reported relatively low levels of lameness (1.3%) and hoof overgrowth (6.27%) in semi-extensive systems. In the present study, higher but still relatively low levels of mild lameness were observed in semi-extensive and semi-intensive systems (6.4% and 5.01%, respectively). In contrast, Parés et al. [8] found that most farms (66%) reported no lameness, and when present, the proportion of affected animals was typically very low (<1%). Furthermore, Marcone et al. [33] identified a positive association between hoof overgrowth and fleece dirtiness, suggesting shared environmental risk factors such as moisture and hygiene. Consistent with this, the present findings showed higher levels of both fleece dirtiness and hoof overgrowth in semi-extensive and semi-intensive systems. These results support the notion that animals in more intensive systems may be more vulnerable to locomotor disorders, likely due to higher stocking densities, increased moisture accumulation, and greater pathogen exposure [30]. The production system also influences the epidemiology and primary aetiological factors of foot-related lameness in dairy sheep. In the UK, footrot, primarily caused by *Dichelobacter nodosus*, accounts for the majority of lameness cases and is frequently associated with interdigital dermatitis [32]. Other important causes include contagious ovine digital dermatitis, ovine interdigital dermatitis, white line disease, and pedal joint abscess. The principal pathogens involved are *Dichelobacter nodosus*, *Fusobacterium necrophorum*, *Treponema* spp., and *Actinomyces pyogenes* [30].

Eye condition proved to be a robust welfare indicator, demonstrating high interobserver reliability, as well as strong sensitivity and specificity. Ocular abnormalities were clearly identifiable under field conditions [4]. Parés et al. [8] reported a higher prevalence of ocular discharge during winter and spring compared with autumn and summer, as well as a tendency for ocular and nasal discharge and respiratory signs to be more frequent in semi-extensive systems. In contrast, ocular discharge was not observed in the present study, and nasal discharge showed a very low prevalence. This discrepancy may be attributed to differences in climatic conditions, seasonal timing of data collection, or improved respiratory management practices on the surveyed farms. Regarding udder health, Marcone et al. [33] identified mastitis as one of the most relevant welfare indicators from farmers’ perspectives, whereas docked tails were considered of minor importance. In the present study, mastitis was recorded but at a relatively low prevalence. Although the highest proportion was observed in semi-extensive farms, differences between systems were not statistically significant (*p* > 0.05). Conversely, semi-intensive farms showed the highest overall prevalence of docked tails, while short-docked tails were most frequent in semi-extensive systems (*p* < 0.05). Given the limited evidence of direct welfare benefits and the potential for pain-related consequences, these findings warrant careful ethical and management consideration. Tail docking practices vary depending on geographic conditions, breed characteristics, and husbandry systems [34]. Furthermore, tail docking is a traditional and painful practice that is often carried out routinely without considering the discomfort it causes. However, the benefits of this practice should be evaluated on a case-by-case basis, and pain-reduction strategies should always be implemented alongside it [35].

The overall prevalence of lesions in the present study was low. However, minor chest lesions were significantly more common in semi-extensive farms (3.07%; *p* < 0.05). The low prevalence of lesions in other body regions is consistent with findings by Koçak et al. [22], who reported low frequencies of major lesions. Similarly, Parés et al. [8] found no significant effects of intensification level, farm size, breed, or season on lesion prevalence, with mean values below 5% per farm. Farmers operating within extensive systems reported a higher frequency of fasciolosis and other parasitic infections. Additionally, cases of sarcoptic mange were reported on one extensive and one semi-extensive farm. Helminthosis is recognised as an important clinical condition associated with internal parasites in sheep production [34]. Parés et al. [8] also reported a significantly higher prevalence of parasites in spring (*p* < 0.01), with some farms showing extremely high levels of ectoparasite infestation. Both free-range and pasture-based systems are exposed to gastrointestinal nematodes, cestodes, flukes, and lungworms, which can negatively affect productivity and health. Therefore, inadequate parasite control may lead to both health and economic losses [34].

Behavioural observation represents one of the most informative approaches for assessing animal welfare, as it enables early detection of distress, disease, or environmental inadequacies. Manenti et al. [36] emphasised that behaviour is a key indicator of animal welfare. Incorporating behavioural knowledge into training programmes for veterinarians and farmers can improve handling practices, reduce stress, and prevent the development of abnormal behaviours. In the present study, sheep from extensive farms showed greater sociability, whereas those from semi-extensive and semi-intensive systems were more frequently described as alert or fearful. Selective breeding for animals that better tolerate reduced handling may represent a practical long-term strategy [26], particularly in systems where consistent positive habituation is difficult to achieve. Sheep possess various genetic adaptations that help them survive in extreme environments. These adaptations are shaped by natural selection and the genetic variability found in key adaptive genes. Such genetic differences are essential for allowing populations to thrive in specific ecological niches as they impact the effectiveness of their adaptations [37]. There is also evidence that genetics influences several aspects of sheep behaviour. This includes mother-offspring interactions, social behaviour, feeding habits, temperament, adaptation to climate, disease resistance, and overall survival [38]. Conversely, limited human contact may increase reactivity to routine procedures, underscoring the significance of the human–animal bond. Regular, calm interactions with caretakers can promote habituation, reduce fear responses, and contribute to positive emotional states [23]. However, negative interactions or a lack of prior human contact may exacerbate stress responses. Overall, the human–animal relationship remains a critical component of sheep welfare, emphasising the need for management strategies that support both biological and behavioural needs across production systems [39].

## 5. Conclusions

The present study demonstrates that sheep welfare is determined not only by production system classification but also by the interaction of multiple factors, including management quality, environmental conditions, genetic adaptation, and preventive health strategies. Each system has distinct welfare vulnerabilities and advantages. In particular, semi-intensive systems demonstrate benefits in structured health and feeding management, while extensive systems promote better behavioural expression and resilience. Key areas of potential welfare issues were identified through indicators such as fleece condition, lameness, tail hygiene, and behavioural profiles (e.g., fearfulness, sociability, and inquisitiveness). These findings emphasise the importance of targeted management practices. To improve welfare outcomes, it is necessary to ensure regular health monitoring, maintain clean and dry housing conditions, and reduce practices associated with fleece damage and tail docking. Appropriate handling strategies are especially important for fearful animals, while stable group housing supports positive social behaviour. Future research should focus on the interactions between breed adaptation, management intensity, and human–animal relationships to further optimise welfare strategies in small ruminant production systems.

## Figures and Tables

**Table 1:** Characteristics of sheep farms.

PARAMETER		PRODUCTION SYSTEMS		
		Extensive	Semi-Extensive	Semi-Intensive
No. of ewes (mean ± sd)		65.8 ± 20.31	146.9 ± 114.97	245.3 ± 254.33
No. of rams (mean ± sd)		3.1 ± 2.51	5.9 ± 3.73	10.7 ± 13.01
No. of lambs (mean ± sd)		81.4 ± 25.49	178.7 ± 122.33	273.9 ± 249.2
No. of adult ewes in the assessed pen/pens (mean ± sd)		36 ± 6.45	47.5 ± 16.52	54.3 ± 21.21
Access to pasture (% of farms)		100	40	80
Local breeds (% of the farms) (Sjenička sheep; Lipska and Vitoroga Pramenka)		90	30	20
Foreign breeds (% of farms) (Romanov, Île-de-France, Württemberg, and Suffolk)		10	70	80
The purpose of sheep farming (% of farms)	meat	50	50	60
	dual-purpose (meat/milk)	30	20	10
	dual purpose (meat/wool)	10	10	0
	dual-purpose (meat/breeding stock/embryo transfer)	10	20	30

**Table 2:** Resource-based indicators on investigated farms.

INDICATOR		PRODUCTION SYSTEMS			*p*
		Extensive	Semi-Extensive	Semi-Intensive	
No. of sheds or housing for sheep (mean ± sd)		1.6 ± 0.97	2.1 ± 1.20	2.5 ± 1.51	*ns*
Presence of exterior pen (% of farms)		80	70	80	*ns*
Number of animals per square meter (mean ± sd)		1.34 ± 0.68	1.46 ± 0.55	1.78 ± 0.51	*ns*
Bedding material (% of each type)		straw: 100 corncob bedding: 0	straw: 80 corncob bedding: 20	straw: 90 corncob bedding: 10	*
Sufficient bedding (% of farms)		low: 30 medium: 50 high: 20	low: 40 medium: 40 high: 20	clean: 40 partly dirty: 50 dirty: 10	*
Bedding cleanliness (% of farms)		clean: 20 partly dirty: 50 dirty: 30	clean: 20 partly dirty: 40 dirty: 40	clean: 40 partly dirty: 50 dirty: 10	*
Outdoor ewes’ accessible shelter (% of farms)		20	30	30	*ns*
Drinker availability (% of farms)		bucket/trough: 100 automatic drinker: 0 natural water source: 40	bucket/trough: 100 automatic drinker: 10 natural water source: 20	bucket/trough: 70 automatic drinker: 80 natural water source: 0	*
Number of functioning water places (mean ± sd)		5.1 ± 4.58 ^bc^	11.4 ± 11.37	17.7 ± 13.19	*
Drinker cleanliness (% of the farms)	bucket/trough	clean: 10 partly dirty: 70 dirty: 20	clean:20 partly dirty: 50 dirty: 30	clean: 20 partly dirty: 20 dirty: 30	*
	automatic drinker	clean: *na* partly dirty: *na* dirty: *na*	clean: 10 partly dirty: 0 dirty: 0	clean: 60 partly dirty: 20 dirty: 0	*ns*
	natural water source	clean: 40 partly dirty: 0 dirty: 0	clean: 20 partly dirty: 0 dirty: 0	clean: *na* partly dirty: *na* dirty: *na*	*

*na*—not applicable; P-statistics probability; *: statistically significant at *p* < 0.05; *ns*—not-significant. ^b,c^ Values with different superscript letters within a row are significantly different (Tukey test, *p* < 0.05).

**Table 3:** Management-based indicators on investigated farms.

INDICATOR	PRODUCTION SYSTEMS			*p*
	Extensive	Semi-Extensive	Semi-Intensive	
Written health plan (% of farms)	20	50	100	*
The most common diseases on the farms (% of farms)	lameness: 20 mastitis: 10 fasciolosis: 30 sarcoptic mange: 10 pneumonia: 0 other parasitic infections: 10 postpartum prolapse: 0	lameness: 40 mastitis: 30 fasciolosis: 20 darcoptic mange: 10 pneumonia: 20 other parasitic infections: 10 postpartum prolapse: 20	lameness: 10 mastitis: 30 fasciolosis: 0 sarcoptic mange: 10 pneumonia: 20 other parasitic infections: 20 postpartum prolapse: 20	*
Combined grazing (% of the farms)	80	30	20	*
Lamb castration	80	20	0	*
Lambs’ age at castration (% of the farms)	>7 days of age: 20 >3 months of age: 60	>7 days of age: 10 >3 months of age: 10	>7 days of age: 0 >3 months of age: 0	*
Frequency of bedding replacement (% of farms)	several times a month: 10 every 2 months: 0 every 3 months: 10 every 6 months: 10 once a year: 70	several times a month: 0 every 2 months: 10 every 3 months: 40 every 6 months: 20 once a year: 30	several times a month: 20 every 2 months: 10 every 3 months: 50 every 6 months: 10 once a year: 10	*
Regular hoof inspection (% of farms)	20	70	80	*
Frequency of claw trimming (% of farms)	as needed: 80 once a year: 20 twice a year: 0	as needed: 50 once a year: 30 twice a year: 20	as needed: 30 once a year: 40 twice a year: 30	*
Nutrition type of forages (% of the farms)	alfalfa hay: 40 meadow hay: 80 corn silage: 0 pasture: 100	alfalfa hay: 100 meadow hay: 60 corn silage: 20 pasture: 40	alfalfa hay: 100 meadow hay: 70 corn silage: 40 pasture: 80	*
Concentrate mixtures (% of the farms)	commercial concentrate mixtures: 0 farm-produced concentrates: 100	commercial concentrate mixtures: 40 farm-produced concentrates: 90	commercial concentrate mixtures: 40 farm-produced concentrates: 80	*
Amount of concentrate mixture (g/head) (mean ± sd)	280 ± 58.69 ^bc^	575 ± 256.31	665 ± 270.85	*
Access to grazing (days/year) (mean ± sd)	252 ± 35.21 ^bc^	105 ± 135.83	148.5 ± 110.18	*

P-statistics probability; *: statistically significant at *p* < 0.05. ^b,c^ Values with different superscript letters within a row are significantly different (Tukey test, *p* < 0.05).

**Table 4:** Animal-based indicators (individual level).

INDICATOR		PRODUCTION SYSTEMS		
		Extensive	Semi-Extensive	Semi-Intensive
Body condition score	very thin	2.95 ± 2.82 ^bc^	5.82 ± 3.68	5.89 ± 4.07
	very fat	3.72 ± 4.78	6.07 ± 4.52	6.39 ± 3.14
Fleece cleanliness	fleece is dry or damp, with light soiling	8.69 ± 2.25	9.21 ± 0.66	9.73 ± 1.34
	fleece is wet, soiled with mud or faeces	3.42 ± 1.85	4.29 ± 1.75 ^c^	2.65 ± 1.84
	Fleece is very wet, heavily soiled	0	0.32 ± 0.32 ^c^	1.23 ± 1.23
Fleece qualities	minor loss	9.55 ± 3.23	8.69 ± 1.65	8.50 ± 2.46
	major loss	6.76 ± 3.75 ^c^	5.04 ± 2.42 ^c^	1.02 ± 0.60
Head, neck, ear lesions	head minor	0.25 ± 0.25	1.02 ± 0.55	0.23 ± 0.23
	head major	0	0.40 ± 0.27	0.86 ± 0.64
	ears minor	0	1.69 ± 1.27	0
	neck minor	0	0	1.00 ± 1.00
Body lesions	chest minor	0	3.07 ± 1.65 ^c^	0.44 ± 0.44
	chest major	0.25 ± 0.25	0.46 ± 0.32	0.59 ± 0.48
	udder lesions	0.25 ± 0.25 ^b^	0.57 ± 0.45	1.29 ± 5.43
Lameness	mild	2.58 ± 1.35 ^bc^	6.40 ± 1.76	5.01 ± 1.36
	severe	0.50 ± 0.33	0.62 ± 0.46	1.29 ± 0.72
Mastitis	subclinical	0.66 ± 0.43	1.19 ± 0.81	0.44 ± 0.44
	clinical	0.56 ± 0.38	0.64 ± 0.43	0.49 ± 0.17
Hoof overgrowth		1.45 ± 0.48 ^bc^	4.64 ± 0.73	5.88 ± 1.60
Leg injuries		0.25 ± 0.25 ^b^	1.87 ± 1.01 ^c^	0.12 ± 0.12
Nasal discharge		3.77 ± 1.57 ^bc^	1.54 ± 0.99	0.64 ± 0.43

^b,c^ Values with different superscript letters within a row are significantly different (Tukey test, *p* < 0.05).

## Data Availability

The original contributions presented in this study are included in the article. Further inquiries can be directed to the corresponding author.

## Abbreviations

The following abbreviations are used in this manuscript:

AWIN	Animal Welfare Indicators
PCA	Principal Component Analysis
BCS	Body condition score
